# Vaccination of influenza a virus decreases transmission rates in pigs

**DOI:** 10.1186/1297-9716-42-120

**Published:** 2011-12-20

**Authors:** Anna Romagosa, Matt Allerson, Marie Gramer, Han Soo Joo, John Deen, Susan Detmer, Montserrat Torremorell

**Affiliations:** 1 Department of Veterinary Population Medicine, College of Veterinary Medicine, University of Minnesota, 385 Animal Science/Veterinary Medicine building, 1988 Fitch Avenue, St Paul, MN 55108, USA

## Abstract

Limited information is available on the transmission and spread of influenza virus in pig populations with differing immune statuses. In this study we assessed differences in transmission patterns and quantified the spread of a triple reassortant H1N1 influenza virus in naïve and vaccinated pig populations by estimating the reproduction ratio (*R*) of infection (i.e. the number of secondary infections caused by an infectious individual) using a deterministic Susceptible-Infectious-Recovered (SIR) model, fitted on experimental data. One hundred and ten pigs were distributed in ten isolated rooms as follows: (i) non-vaccinated (NV), (ii) vaccinated with a heterologous vaccine (HE), and (iii) vaccinated with a homologous inactivated vaccine (HO). The study was run with multiple replicates and for each replicate, an infected non-vaccinated pig was placed with 10 contact pigs for two weeks and transmission of influenza evaluated daily by analyzing individual nasal swabs by RT-PCR. A statistically significant difference between *R *estimates was observed between vaccinated and non-vaccinated pigs (*p *< 0.05). A statistically significant reduction in transmission was observed in the vaccinated groups where *R *(95%CI) was 1 (0.39-2.09) and 0 for the HE and the HO groups respectively, compared to an *R*_o _value of 10.66 (6.57-16.46) in NV pigs (*p *< 0.05). Transmission in the HE group was delayed and variable when compared to the NV group and transmission could not be detected in the HO group. Results from this study indicate that influenza vaccines can be used to decrease susceptibility to influenza infection and decrease influenza transmission.

## Introduction

Influenza in pigs is a highly contagious viral disease of the respiratory tract. Influenza is currently endemic in most swine populations around the world, and the virus tends to spread easily in susceptible populations [1-3]. Many factors contribute to the severity of the disease including age, viral strain, concurrent infections, and immune status of the animals [3-5].

With the detection of new influenza subtypes in the last decade (i.e. H1N1, H1N2 and H3N2 triple reassortant viruses) [6-8] in pigs and the recent appearance of the 2009 pandemic H1N1, both human and animal health officials have paid greater attention to flu in pigs due to the role that pigs play in inter-species transmission [9]. The control of influenza in pigs is often accomplished by the use of vaccines [10]. Both inactivated licensed commercial vaccines and autogenous licensed inactivated vaccines are commonly used in pigs. Commercial vaccines confer protection against flu infection and disease presentation but often this protection is only partial [11-13]. Commercial vaccines usually include one or more isolates representative of the strains in a region but they may not always confer protection against the isolate infecting a specific farm or population. On the other hand, autogenous vaccines may be prepared with the isolate or isolates recovered from a specific production system and restricted to use in only that system. These vaccines have gained popularity in the US in the past few years. Although vaccination can result in the reduction of clinical signs and virus shedding, limited information is available on the effect that vaccination has on population susceptibility, the spread of infection and how vaccination may prevent transmission to other species [14].

Transmission experiments and mathematical models have been used to quantify vaccine-induced reduction in the spread of *Mycoplasma hyopneumoniae*, pseudorabies virus, classical swine fever, *Actinobacillus pleuropneumoniae*, encephalomyocarditis virus (EMCV), foot and mouth disease (FMDV), porcine reproductive and respiratory syndrome virus (PRRSV), hepatitis E virus, and porcine circovirus type 2 (PCV-2) in pigs [15-23]. In order to quantify transmission of a pathogen, a key parameter is the reproduction ratio (*R*) of the infection which is defined as the average number of secondary cases caused by an infectious individual in a population during its entire infectious period [24,25]. When *R *is greater than 1, an infection can spread in a population but if *R *is less than 1, the infection will die out within a population. The estimation of *R *can provide important information about the potential for transmission of infection, the dynamics of infection at the population level, and the impact of disease control strategies [15,26,27].

The reproduction ratio has been assessed for influenza A virus in humans, birds, and horses [28-33], but *R *has not been reported for influenza virus A in pigs. In this study, a deterministic SIR model (Susceptible-Infected-Recovered/Removed) was used to compare transmission parameters between a non-vaccinated population and vaccinated populations of pigs following the introduction of a non-vaccinated, infected pig with a triple reassortant H1N1 influenza A virus. The introduction of infected pigs into populations is one of the primary modes of influenza virus transmission in field settings and this study mimics a similar scenario. Specifically we aimed at assessing the effect of vaccination on pig susceptibility to infection. Since different vaccines containing inactivated viruses that were either homologous or heterologous to the challenge virus were used in this study, an additional comparison could be made between vaccine types. Results from this study provide relevant information on the use of vaccination to control influenza transmission, and highlight the implications of partial protection may have in transmission dynamics and risk of infection.

## Materials and methods

### Animals and animal housing

One hundred and ten, three-week-old cross-bred pigs from a specific-pathogen-free (SPF) herd were obtained. Pigs were free of infection with influenza virus, PRRS virus, and *M. hyopneumoniae*. The sows had not been vaccinated against influenza virus and all piglets were screened at the herd of origin for influenza antibodies using hemagglutination inhibition (HI) test and influenza A Multiscreen enzyme linked immunosorbent assay (ELISA, IDEXX FlockChek™ AI MultiS-Screen Ab Test Kit, IDEXX Lab., Westbrook, ME, USA) prior to the start of the study.

Pigs were randomly distributed into ten groups of eleven pigs each and placed in separate isolation rooms located at the University of Minnesota Animal Research Facility (St Paul, MN, USA). Pigs were assigned to 3 different treatment groups as follows: (i) non-vaccinated control (NV); (ii) vaccinated with a commercially licensed, heterologous vaccine (HE); and (iii) vaccinated with an experimental, homologous vaccine (HO). There were 3 replicates for the NV and the HO treatments and 4 replicates for the HE treatment. Space allowance was 6.3 square feet per pig (0.58 m^2^) and the pigs were fed on the floor ad libitum and with free access to water. Pigs were cared for according to University of Minnesota IACUC protocol number 0908A71965.

### Experimental design

Nasal swabs and blood samples were collected from all pigs upon arrival to the research facility and tested by real time reverse transcriptase PCR (RT-PCR), and for antibodies by HI and influenza A Multiscreen ELISA. Pigs were also injected once with an antibiotic per label instructions in order to reduce bacterial contaminants prior to the start of the study (Ceftiofur crystalline free acid, 5.0 mg/kg body weight Excede^®^, Pfizer Animal Health, NY, USA).

Twenty-four hours post arrival all pigs were vaccinated according to their treatment group. Pigs in the HE group received 2 mL intramuscularly (IM) of a commercial licensed influenza vaccine (FluSure XP^®^, Pfizer Animal Health, New York, USA). Pigs in the HO group were similarly vaccinated with a homologous, inactivated vaccine containing the same viral isolate as the challenge virus. Each vaccination was repeated two weeks later. The pigs in the NV group were injected with 2 mL of sterile saline solution IM in the neck at 2 weeks interval. In each room 1 of the 11 pigs (designated "seeder") was left unvaccinated to be intratracheally and intranasally challenged with influenza and serve as a source of infection for the other pigs in the group.

Thirteen days after the second vaccination, nasal swabs and blood samples were taken from all pigs, and the seeder pigs were moved to a separate room for challenge. At 48 h post challenge, the seeder pigs were placed back to each room in contact with their original pen mates (1 seeder pig/group/replicate) until the termination of the study. To determine transmission, all pigs were sampled daily by taking nasal swabs and observed daily for presence of clinical signs consistent with influenza. The transmission experiment ended at 14 days post contact (dpc) or when all 10 contact pigs in a room became influenza virus positive. At that point, pigs were humanely euthanized with an intravenous lethal dose of pentobarbital at the prescribed amount of 100 mg/kg (Fatal-Plus Solution^®^, 250 mL, Vortech Pharmaceuticals, Dearborn, MI, USA).

### Challenge virus and vaccine preparation

A triple reassortant H1N1 strain A/Sw/IA/00239/04 (IA04) belonging to the β cluster used in previous studies [34-36] and isolated from field samples at the University of Minnesota Veterinary Diagnostic Laboratory was used for challenge. The IA04 influenza virus was grown in bulk quantities using Madin-Darby canine kidney (MDCK) cells using standard procedures [37].

For preparation of the homologous vaccine, the same virus IA04 was adjusted to an HA titer of 1:128/0.1 mL at the time of inactivation by the addition of formalin at a final concentration of 0.1%. The formalized virus was mixed with an adjuvant mixture of mineral oil (9 parts) and emulsifier (1 part; equal volumes of Span 85 and Tween 85) in a 1:1 ratio and sonicated at 25W for 2-3 min.

The heterologous vaccine contained three distinct inactivated influenza isolates and an adjuvant: A/Swine/NorthCarolina/031/05 (H1N1), A/Swine/Missouri/069/05 (H3N2), and A/Swine/Iowa/110600/00 (H1N1). The H1N1 vaccine strains belonged to the γ and δ groups and were genetically distinct from the challenge strain. A/Swine/Iowa/110600/00 (γ) and A/Swine/North Carolina/031/05 (δ) shared 92.2% and 66.8% HA nucleotide similarity respectively, with IA04 (Mega 4 with Clustal W alignment, Nucleotide Kimura's). Serologic cross-reactivity existed between the licensed vaccine strains and challenge strain but was variable [35].

### Virus inoculation/seeder pigs

Seeder pigs were infected intratracheally and intranasally with a total of 2 mL of the IA04 H1N1 challenge virus at a titer of 1 × 10^6 ^tissue culture infectious dose (TCID)50/mL. Before the inoculation, all piglets were sedated by an intramuscular injection of a dissociative anesthetic at the recommended dose of 6.6 mg/kg (Telazol^®^, Fort Dodge Animal Health, Fort Dodge, IA, USA).

Success of inoculation in the seeder pigs was confirmed by positive influenza A RT-PCR from nasal swabs at 24 h and at 48 h post inoculation. Viral isolation and titration was conducted from nasal swabs at 48 h post inoculation.

Researchers conducting the experimental infection wore N-95 respirator masks, protective glasses and gloves when performing this procedure and throughout the entire study when they were accessing the pigs.

### Transmission chain

Each transmission experiment consisted of ten susceptible (contacts) and one infectious pig (seeder) per group. The initial infectious seeder pig in all groups was unvaccinated and challenged intratracheally and intranasally with an H1N1 influenza virus. A pig was considered infected and infectious when the virus could be detected by RT-PCR from nasal swabs. Transmission experiments ended at 14 dpc with the seeder pig, or when all the contact pigs in a replicate became infected.

### Sample processing and diagnostic tests

#### Nasal swabs

Nasal swabs were collected daily from individual pigs using rayon-tipped swab applicators with Stuart's medium (BBL CultureSwab™ liquid, Stuart single plastic applicator/Becton, Dickinson and Com., Sparks, Maryland 21152, USA). After sample collection, each nasal swab was suspended in 2 mL of minimum essential medium (MEM, Mediatech Inc., Manassas, VA, USA) supplemented with 4% bovine serum albumin (BSA, Sigma-Aldrich Co., St. Louis, MO, USA) prior to processing for RT-PCR. The viral RNA was extracted using the magnetic particle processor procedure (MagMAX™ Viral RNA Isolation Kit, Applied Biosystems, USA) and subsequently tested using the procedure provided by the USDA-NVSL for detection of influenza A virus Matrix gene by RT-PCR [38]. The minimum detection limit of influenza virus by this method is 10^1 ^TCID_50_/mL.

Virus isolation and titration was performed only from the nasal swabs collected from the seeder pigs prior to commingling, and from tissues at necropsy on MDCK cell monolayers [37]. Supernatants showing cytopathic effect (CPE) were titrated using ten-fold serial dilution and expressed as a log 10 TCID_50_/mL calculated by the Spearman-Kärber method [39].

#### Blood Samples

Blood samples were collected using venipucture of the jugular vein. After collection serum was separated and stored at -20°C. Sera were subsequently analyzed for the detection of influenza virus antibodies by HI and influenza A Multiscreen ELISA. HI tests were performed following standard procedures [40]. Samples were tested by HI against the challenge strain (IA04), the licensed commercial vaccine isolates γ and δ (A/Sw/IA/110600/00 and A/Sw/NC/031/2005, respectively), and H3N2 (A/Sw/MO/069/2005 H3N2) at arrival, thirteen days after the second vaccine, and at necropsy. Additionally, all sera were tested using the Influenza A Multiscreen ELISA following manufacturer's protocols. The Influenza A Multiscreen ELISA measures antibodies directed against the nucleoprotein (NP) of influenza A viruses.

#### Tissues

At necropsy, lung affected by pneumonia was recorded for each pig. Lesions were photographed, sketched on a standard diagram, and the proportion of affected lung assessed [41]. In addition, tissues from lung lobes of each animal were collected for viral detection by RT-PCR.

For histopathology, the tissues were fixed in 10% buffered formalin and paraffin-embedded by standard techniques. Tissue sections were stained with hematoxylin and eosin and examined microscopically for histopathologic changes. Lung sections were examined for bronchiolar epithelial changes, and peribronchiolar and alveolar inflammation. Lung sections were given a score from 0-3 to reflect the severity of bronchial epithelial injury based on previously described methods [42]. The lung sections were scored according to the following criteria: 0, no significant lesions; 1, a few airways affected with bronchiolar epithelial damage and light peribronchiolar lymphocytic cuffing often accompanied by mild focal interstitial pneumonia; 1.5, more than a few airways affected (up to 25%) often with mild focal interstitial pneumonia; 2, 50% airways affected often with interstitial pneumonia; 2.5, approximately 75% airways affected, usually with significant interstitial pneumonia; 3, greater than 75% airways affected, usually with interstitial pneumonia. Additionally, the proportion of airways affected in ten microscopic fields at 20 X magnification was also recorded. A single pathologist examined all slides and was blinded to the treatment groups.

### Clinical signs

Clinical signs consistent with influenza were recorded from day 1 to day 4 post arrival, during the 3 days after the second vaccination, and daily from 0 dpc to the end of experiment (14 dpc). Clinical signs of cough, dyspnea, sneezing, nasal discharge and lethargy were recorded for a period of 10 min/observation day in each room. Rectal temperatures were recorded daily from 1 day prior to commingling until necropsy. Temperatures ≥ 40°C were considered a significant febrile response.

### Statistical methods

Results from clinical signs, antibody titers, and lung lesions were combined for each treatment group, resulting in 30 contact pigs in the control and homologous groups, and 40 contact pigs in the heterologous group. The seeder pig in each replicate was excluded from these analyses.

To compare antibody responses before exposure and at necropsy, log-transformed results (HI antibody titers) and influenza A Multiscreen ELISA mean values were analyzed using a paired t-test and results were considered statistically significant at *p *< 0.05. Clinical signs were evaluated as a binomial response (0 = absence/1 = presence) by a Fisher's exact test and by a Generalized Estimating Equations (GEE) approach [43]. Presence or absence of each clinical sign for each pig on a daily basis was scored as 1 or 0 respectively, and analyzed as repeated measures for categorical data and clustered responses in each group. Clinical signs were compared between all pigs in the HE and NV groups via an odds ratio.

Means and standard deviations from macroscopic and microscopic lung lesion scores were analyzed using ANOVA. The response variables that had a statistically significant effect (*p *≤ 0.05) by treatment group were further analyzed through pair-wise comparisons using the Tukey-Kramer test. The analyses were performed using SAS (SAS System, SAS Inst., Cary, North Carolina, v 9.2).

#### Survival curves and infectious period (IP)

The cumulative percentage of contact pigs infected per day in each group was compared by Kaplan-Meier survival curves and log-Rank test using SAS and R (SAS System, SAS Inst., Cary, North Carolina, v 9.2 and R Development Core Team (2009), R: A language and environment for statistical computing. R Foundation for Statistical Computing, Vienna, Austria). Differences between curves were further evaluated by pair wise comparison using the Tukey-Kramer test. Pigs remaining influenza PCR negative at the end of the study were censored.

The survival curves were also used to calculate the infectious period. The infectious period was defined as the time between the first and the last day that virus could be detected from nasal swabs in a pig. Differences in the infectious period and the average number of days between contact exposure and the first pig detected positive were calculated using the log-Rank test with significant differences considered at *p *value < 0.05.

#### Estimation of the reproduction ratios (R)

The infection status of susceptible (S), infectious (I), and recovered/removed (r), was determined on a daily basis for all pigs. Transition of pigs from the S state to I state, and from I state to r state was described by a deterministic SIR model [44]. For each time interval Δt (the interval between two consecutive samplings (i.e. one day)), a pig was considered I if influenza virus was detected by RT- PCR on nasal swabs, and it was considered S if influenza virus was not detected positive by RT-PCR. When transmission occurred in a pen, S pigs decreased by one (S-1) whereas the number of I pigs increased by one (I+1). The transition from S to I occurs according to the probability given by γ = β S (t) I (t) Δt, with the infection or transmission parameter β denoting the rate at which a randomly chosen animal had infectious contacts in the interval Δt.

To estimate the transmission parameter β, a generalized linear model (GLM) with a complementary log-log link function and log I Δt/N as the offset variable (number of infectious pigs/total number of pigs) was used to calculate the estimates of the transmission parameter β by day (Δt = 1) [45]. Knowing the number of susceptible contact pigs and the number of infectious pigs at the start of each period Δt (S_t-1_, I_t-1 _), the number of new infections that appeared at the end of each period (C_t_), and the total number of animals in each period (N), the probability that a pig became infected was 1-e^-IβΔt/N ^and the number of animals infected during each time period was E(C)= S(1- e^-IβΔt/N^) [18].

The logistic regression model cannot provide a direct estimate of the transmission rate β, but with an alternative transformation known as the complementary log-log we calculated β from the equation log [-log (1-E(C)/S)] = log (β) + log (IΔt/N). This model is similar to the linear model as follows: log [-log (1-E(C)/S)] = βo + β_1_X where the intercept coefficient βo = log (β), with corresponding β_1 _= 1 including the predictor X = log (IΔt/N) as a fixed offset. With the transformation of log (β), the transmission parameter β could be estimated. The model entails some assumptions: (i) all susceptible animals are equally susceptible; (ii) all infected animals are equally infectious; (iii) each infected pig poses an independent risk of infection to each susceptible pig. Differences between β values were compared using chi-square comparisons and differences were considered statistically significant at *p *< 0.05.

Using the daily transmission rate β and the infectious period of the contact infected pigs, the reproduction ratio *R *of the infection was calculated for each replicate in each group, as well as the overall *R *for each group of treatment after the replicates were combined. Statistically significant differences between *R *estimates were based on the existence of non-overlapping 95% confidence intervals (C.I.'s). If a pig died during the study, it was considered censored and N became N-1. All analyses were carried out in SAS (SAS System, SAS Inst., Cary, North Carolina, v 9.2).

In our study the GLM method was used because the NV group had zero susceptible animals when the infection process ended, and in that situation the effect of vaccination only can be tested by GLM analysis [25]. In addition, the GLM method can be used to analyze data of heterogeneous populations as is the case of our study since contact animals were vaccinated and inoculated animals were not.

#### Stochastic SIR model

A stochastic SIR model was developed using the transmission and recovery parameters obtained from the previously described deterministic model and experimental study (parameters listed in Table 1). For all stochastic models, the initial number of infectious, susceptible, and recovered pigs was 1, 10, and 0, similar to that of the experimental study. In the stochastic models two events could occur, transmission (S→I) and recovery (I→R) within the SIR model (S→I→R). The transmission rate (β) was obtained from the deterministic model and the recovery rate (γ) was obtained from the experimental study (the inverse of the infectious period). The rate at which transmission occurred was βSI and the rate at which recovery occurred was γI. Each replicate for which transmission occurred in the experimental study was modeled separately with 10 000 simulations using the parameters obtained from the experimental deterministic model. Each simulation was run until the infectious process ended and a final size of the outbreak could be assessed. The number of new cases (contact infected susceptible individuals) observed for each simulation, given the parameters listed in Table 1, was assessed for each replicate with the initial number of infectious, susceptible, and recovered individuals at 1, 10, and 0, respectively. The proportion and cumulative proportion of 10 000 simulations by the number of new cases for each replicate was displayed in graph format. The direct method of Gillespie was used to model the random events of transmission and recovery [46].

**Table 1:** Transmission parameter estimates and related 95% confidence intervals (CI) for each replicate and overall treatment groups

GROUP	Replicate	IP^1^	β^2 ^(95%CI)	R^3 ^(95%CI)	R^4 ^(95%CI)
NV	1	4.7	1.99 (0.97-3.59)	9.35 (4-18.7)	
NV	2	4.2	4.71 (2.20-9)	19.81 (8.3-41.4)	10.66 (6.57-16.46)^a^
NV	3	4.6	1.85 (0.91-3.29)	8.51 (3.2-18.3)	

HE	1	4	0.12 (0.01-0.56)	0.51 (0.02-2.26)	
HE	2	5	0.36 (0.13-0.77)	1.8 (0.47-3.9)	0.99 (0.39-2.09)^b^
HE	3	3.5	0.09 (0.01-0.4)	0.32 (0.01-3.27)	
HE	4	2.4	0.53 (0.19-1.15)	1.27 (0.21-1.74)	

HO	1	0	2.2310^-6 ^(na-0.52)	0	
HO	2	0	2.2310^-6 ^(na-0.52)	0	0^c^
HO	3	0	2.9410^-6 ^(na-0.52)	0	

The number of initial infectious (Io) and susceptible (So) pigs in each replicate were Io = 1 and So = 10, except for HE 4 where the So = 9.

^1^IP: average duration (days) of the infectious period for the contact pigs.

^2^β: transmission rate per day.

^3^Reproduction ratio R by replicate.

^4^Reproduction ratio R when replicates were combined by group.

^a,b,c^Statistically significant differences (*p *< 0.05).

## Results

### Serology

Pigs were both antibody and influenza virus negative at the start of the study. Table 2 shows the levels of HI antibody titers against the 4 selected flu strains prior to exposure to the seeder pigs and at necropsy. Two weeks after the second vaccination, the homologous vaccine induced robust HI titers against the challenge strain (IA04), but mean reciprocal titers in the HE group were below 1:20. In contrast, the HE group had reciprocal titers above 1:160 against the H1N1 and the H3N2 strains contained in the vaccine (IA00, NC05 and MO05), while the mean reciprocal titers in the HO group were below 1:40. At the end of the experiment, HI titers against the challenge virus remained at about the same levels in the HO group, and although a statistically significant increase in mean titers in the HE group was observed (*p *< 0.05), they still remained below 1:40. In the NV group, all pigs were seronegative (1< 10) before contact and at necropsy, and the lack of seroconversion at necropsy was most likely due to the limited time between infection and necropsy (less than 7 days) [36,47].

**Table 2:** HI titers (reciprocal geometric means) against 4 influenza strains (challenge strain IA04 (β) and the commercial vaccine isolates: XP12H1(γ), XP31H1(δ) and the XP69H3) by group of treatment

HI titers								
Group	IA04		XP12H1		XP31H1		XP69H3	
	before	necropsy	before	necropsy	before	necropsy	before	necropsy
NV	< 10	< 10	< 10	< 10	< 10	< 10	15	12
HE	14^a^	33^b^	307	240	229	82	494	344
HO	297	360	38	57	< 10	< 10	< 10	21

Before contact (- 2 dpc) and at necropsy (i.e. + 7 dpc for the control group/+14 dpc for the vaccinated groups).

^a,b ^Statistically significant differences (*p *< 0.05).

**Figure 1:**
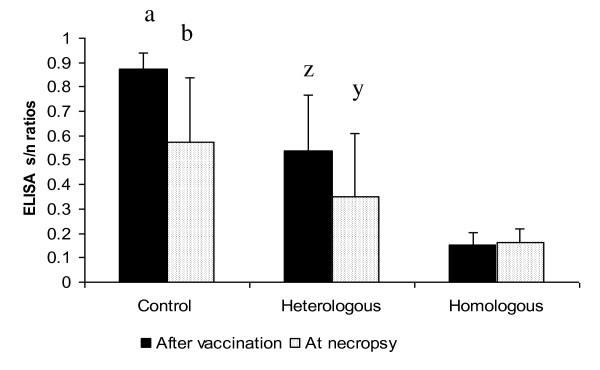
**Paired influenza A Multiscreen ELISA s/n values against the nucleoprotein of influenza virus two weeks after vaccination (prior to exposure) and at necropsy (bars represent the mean of s/n values (± SD) to influenza ■ after vaccination, □ at necropsy**. Positive s/n < 0.673; negative s/n > 0.673. ^a,b and z,y ^Statistically significant differences between paired samples for the NV and HE groups before exposure and at necropsy (p < 0.05).

Mean influenza A Multiscreen ELISA s/n values (± SD) of the contact pigs two days prior to exposure to the seeder pigs were 0.874 ± 0.06 in the NV group, 0.538 ± 0.23 in the HE group, and 0.15 ± 0.05 in the HO group (cut-off: positive < 0.673, negative > 0.673) [48]. At necropsy, the mean s/n responses were 0.576 ± 0.26, 0.351 ± 0.26 and 0.162 ± 0.05 for the NV, the HE and the HO groups respectively. Differences between paired samples in both the NV and the HE groups before contact and at necropsy were statistically significant (*p *< 0.05), (Figure 1).

### Transmission

#### RT-PCR test on nasal swabs

All seeder pigs were RT-PCR positive at 48 h post inoculation and prior to comingling with the contact susceptible pigs. At that time point, the virus titers from nasal swabs in those pigs ranged from 3 × 10^2 ^to 1 × 10^5 ^TCDI_50_/mL.

**Figure 2:**
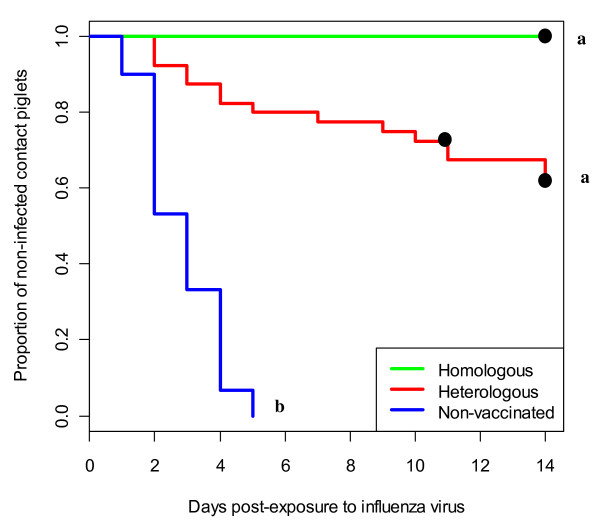
**Time to infection curves for the three treatment groups**. ^a,b^Statistically significant differences (p < 0.05) Difference between vaccine groups (a) is statistically significant at 90% confidence level (p = 0.101) ● Censored data.

All the contact pigs (100%) in the NV group were found RT-PCR positive by 5 dpc, while in the vaccinated groups, 15 out of 40 pigs (37.5%) in the HE group and none of the pigs (0%) in the HO group were positive by RT-PCR (Table 3). Comparison of the cumulative percentages of contact pigs infected per day between treatment groups via the log-Rank test from the Kaplan-Meier survival analysis indicated that the percentage of infected pigs was significantly higher in the NV group than in the vaccinated groups (*p *< 0.0001), and the distributions did not overlap between 90% confidence intervals (Figure 2).

**Table 3:** Number of RT-PCR positive contact pigs, per group of treatment and replicates at the end of the study

Treatment	Replicate	Positive contact pigs	Total number of contact pigs
Non-vaccinated	1	10	10
Control	2	10	10
	3	10	10

Vaccinated	1	1	10
Heterologous	2	5	10
	3	2	9^a^
	4	7	10

	1	0	10
Homologous	2	0	10
	3	0	10

^a ^One of the contact pig was removed from the group at 7 dpc.

#### Infectious period (IP)

The mean length of the infectious periods (i.e. time between the first and the last day that virus could be detected from nasal swabs (± SD)) in the seeder and in contact pigs are shown in Table 4. The seeder pigs remained PCR positive for an average of 5.08 ± 0.6 days post infection (dpi) and no differences were observed among them. The average infectious period for the contact infected NV pigs was 4.5 ± 1.07 days and 3.50 ± 1.84 days for the contact infected pigs in the HE group. The infectious period was 1 day shorter in pigs vaccinated with the HE vaccine than in the NV pigs, but the difference was not statistically significant (*p *= 0.192). The infectious period for the contact infected pigs in the HO group was zero. In addition, the average number of days between exposure and the first pig detected positive was significantly longer in pigs from the HE group (6.87 ± 4.17) compared to the NV group (2.83 ± 1.14) (*p *= 0.0015).

**Table 4:** Infectious period from seeder (S) and contact pigs (C) when all replicates were combined by group

Group		**Number of pigs excreting virus **^**a**^	**Infectious period **^**b **^**(days ± SD)**	**Days ± SD between exposure and start of excretion **^**c**^
NV	S	3/3	5.66 ± 0.58	-
NV	C	30/30	4.50 ± 1.07	2.83 ± 1.14 ^1^
HE	S	4/4	5.25 ± 0.5	-
HE	C	15/40	3.50 ± 1.84	6.87 ± 4.17 ^2^
HO	S	3/3	4.33 ± 1.53	-
HO	C	0/30	0	-

^a^Replicates from the same treatment were pooled.

^b^The average number of days from the first until the last day that influenza virus could be detected from nasal swabs by PCR.

^c^Average number of days between exposure and the first sample detected positive.

^1, 2^Statistically significant differences (p < 0.05).

#### Reproduction Ratio (R)

A summary of the transmission parameter estimates for each replicate and treatment group are displayed in Table 1. In the HE group, one pig was humanely euthanized at 11 dpc for reasons not related to influenza and was censored, yielding a total number of contact pigs in that replicate of 9.

In the NV group, all contact pigs became infected and *R*_0 _(95% confidence interval) was estimated at 10.66 (6.57-16.46). In contrast, the estimate of *R *was 1.00 (0.39-2.09) in the HE group, and 0 in the HO group. A statistically significant reduction in *R *value was observed in the vaccinated groups (*p *< 0.05). The difference in transmission rate (β) was also statistically significant between the NV and HE groups (*p *< 0.0001). Transmission in the HO vaccinated pigs could not be detected in any of the replicates. Transmission in the HE group was observed in all replicates and although *R *was above 1 in two of the four replicates, *R *was not statistically significant below 1. However, there were statistically significant differences between HE transmission rate β replicates with differences observed between replicates 1, 2, 3 versus 4 (*p *< 0.001).

#### Stochastic SIR model

**Figure 3:**
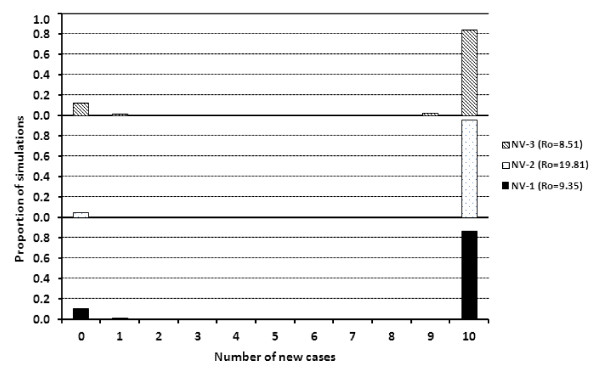
**Number of new cases represented as the proportion of 10 000 simulations from the stochastic SIR model with initial values of (S = 10, I = 1, R = 0) for each NV replicate**.

**Figure 4:**
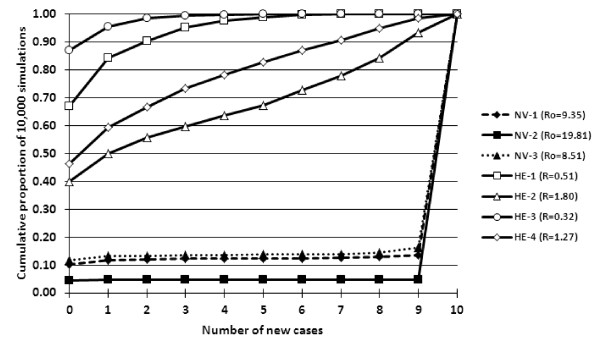
**Cumulative proportion of the number of new cases of 10 000 simulations from the stochastic SIR model with initial values of (S = 10, I = 1, R = 0) for each NV and HE replicate**.

The number of new cases for 10 000 simulations was determined given the parameters listed in Table 1 and the starting values of S = 10, I = 1, and *R *= 0 for each replicate. The stochastic models first highlighted that within this small population, large outbreaks most commonly occurred given the parameters for all NV replicates (Figures 3 and 4). At least 84% of the simulations for each NV replicate yielded 10 contact infected pigs. However, even with transmission and recovery parameters generating an *R*_0 _estimate much greater than 1, simulations yielding no new cases or minor outbreaks also occurred in approximately 15% of the simulations.

**Figure 5:**
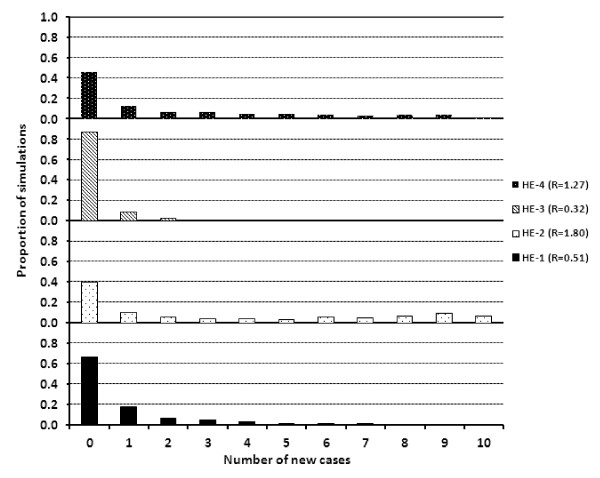
**Number of new cases represented as the proportion of 10 000 simulations from the stochastic SIR model with initial values of (S = 10, I = 1, R = 0) for each HE replicate**.

Stochastic simulations for the HE replicates (Figures 4 and 5) demonstrate that within this small population, major outbreaks were rare and the number of new cases was generally low compared to that of the NV replicates. For all HE replicates, at least 40% of the simulations yielded no new cases and a maximum of 7% of simulations yielded 10 new cases compared to at least 84% of the simulations for the NV replicates. The distribution of the number of new cases was also more variable for the HE replicates compared to that of the NV replicates in which the outbreak was either large or small.

### Clinical signs

Influenza-like clinical signs were mild in all pigs. No pigs died during the study due to influenza infection, and only one contact pig from the HE group had to be removed from the study due to arthritis. The odds of a pig displaying a clinical sign (fever, lethargy, coughing, sneezing, nasal discharge, or dyspnea) during the first week of exposure was significantly lower in the HE group than in NV pigs (OR = 0.37, 95% CI 0.21-0.65, *p *= 0.005).

### Macroscopic and microscopic lung lesions

NV pigs had pneumonia with areas of lung consolidation at necropsy, and the mean (± SD) lung lesion score was 8.86 ± 7.1%. Thirty-one of the 40 pigs in the HE group and sixteen of the 30 pigs in the HO group had some degree of lung consolidation. The mean lung lesion score for the pigs in the HE group was significantly higher (*p *≤ 0.05) than the mean for the pigs in the HO group (5% ± 8 vs. 1.7% ± 5.1) at necropsy. Although the macroscopic lung lesions in the NV group were greater than in the vaccinated groups, comparisons were only performed between treatment groups with the same endpoints.

The lung lesions of the NV pigs had moderate to severe bronchoalveolar pneumonia with attenuation, hyperplasia, moderate numbers of lymphocytes admixed with macrophages in the epithelium, within the intraluminal necrotic cellular debris, and extending into the alveolar spaces. Some lung sections contained occasional bacterial colonies. The overall microscopic lesion mean score was 2.6 ± 0.48 at necropsy indicating significant interstitial pneumonia and more than 75% of the airways affected with bronchial epithelial damage.

The lung lesions of the heterologous vaccine treatment pigs were highly variable ranging from absent to severe. Lesions included moderate numbers of lymphocytes within the hyperplastic epithelium, intraluminal cellular debris and occasional bacterial colonies. One pig had a large abscess in one tissue section and another pig had aspiration pneumonia. The lung lesions of the homologous vaccine treatment pigs were absent to minimal. The lungs of a few pigs had minimal to moderate epithelial attenuation and hyperplasia with small numbers of lymphocytes within and surrounding the epithelium. Many of the tissue sections from all three groups had slight to moderate increases in prominence of the peribronchiolar lymphoid tissue.

The severity of the lung lesions was scored at 1.41 ± 0.49 in the HO group, and at 1.16 ± 0.67 in the HE group, indicating no differences in the severity of the lesions between vaccinated groups (*p *= 0.09). However, differences were statistically significant (*p *< 0.05) between the vaccinated and non-vaccinated groups.

## Discussion

Understanding transmission of influenza and the factors that affect transmission is crucial for designing effective control strategies. Vaccination is by far the most common strategy to prevent flu infections. However, little is known on how vaccination affects transmission within pig populations. The objectives of this study were to quantify the spread of an H1N1 triple reassortant influenza virus in populations of pigs by calculating transmission parameters based on the outcome of transmission experiments, and to assess the effect of vaccine on susceptibility to influenza A virus infection in pigs vaccinated with either a homologous or a heterologous vaccine.

The reproduction ratio *R*, which is the expected average number of secondary infections caused by a typical infectious individual during its entire infectious period [24], is a measure to quantify the transmission and spread of viruses in populations. When infection occurs in an entirely susceptible population, the reproduction ratio is named "basic" reproduction ratio or *R*_0 _[24]. When *R *is greater than 1, an infection can spread in a population but if *R *is less than 1, the infection will die out within a population. *R *estimates can be used to assess the effect of vaccination or other control strategies in populations.

Under the specific conditions of this study and as expected, influenza virus spread quickly among non-vaccinated pigs, and the estimated *R*_0 _in this population was high (10.66, 95% CI 6.57-16.46). The estimated *R *values in the vaccinated groups were significantly different from the values in the NV group. In the HO vaccinated group *R *was estimated at 0, and in the HE group *R *ranged from 0.32 to 1.8, with an overall estimate of 1 (95% CI, 0.39-2.09). However, *R *in the HE group was not statistically significant below 1. Furthermore, statistically significant differences could be observed in the transmission rates (β) between replicates, showing the variability of the pigs' response to influenza vaccination. Despite the fact that transmission was significantly reduced in the HE group, we cannot exclude the possibility that outbreaks of influenza could occur in heterologously vaccinated populations as evidenced by the results of the stochastic model in this study and the modeling estimates presented by de Jong et al. [49]. On the other hand, transmission of the virus could not be detected in the HO vaccinated pigs during the study and *R *values in all replicates were significantly below 1, suggesting that infection spread in a population vaccinated with a homologous vaccine could be prevented.

In our experiment, a SIR model (Susceptible-Infective-Removed (or Recovered)) was used to describe influenza transmission in pigs because the disease confers immunity against re-infection with a homologous strain [44]. Basically, three different methods have been used to calculate the infection parameters based upon experimental data: Generalized Linear Models (GLM) [45,49], Martingale Estimations [16], and Maximal Likelihood Estimation based on the final size of an outbreak (FS) [32,50]. The selection of one of the three approaches depends on the data available and the intensity of the sampling. We focused on quantifying the effect of vaccination on the susceptibility of contact pigs to influenza infection instead of evaluating the effect on infectivity of vaccinated pigs. Transmission depends on both the infectivity of the infected individuals and the susceptibility of the contact pigs. In our study, we predicted that vaccination of the seeder pigs would have influenced the number of excreted influenza A viruses as shown in previous studies [30,35], and therefore we would not have been able to replicate conditions of transmission relevant to field situations where introduction of shedding animals in vaccinated populations is common. Nevertheless further studies are needed to determine the effect of excretion on the infectivity of influenza vaccinated pigs.

Other experimental transmission studies with H7N7 avian influenza virus, PRRSV and Aujeszky's disease were not able to correlate the amount of virus excreted by infected pigs with increased values in the transmission parameter β [20,31,51]. In our study, the initial infectiveness was similar in all the groups due to the use of unvaccinated and experimentally inoculated seeder pigs under the same experimental conditions. Also, no statistically significant differences were observed in TCID_50 _values at the time of exposure with the contact pigs and the infectious period was similar for all seeder pigs. Therefore, differences in transmission between groups can be attributed to the decrease in susceptibility of the contact pigs to become infected, and the length of the IP between groups. The infectious period was one day shorter in the HE group than in the NV group and that could play a role in the value of *R *and account for some of the differences observed between replicates. Overall, detection of influenza virus in the HE vaccinated population lasted longer than in the NV population which also highlights the role of partially immune populations as potential sustained reservoirs for influenza infections.

*R*_0 _of influenza virus has been estimated previously in chickens and horses. *R*_0 _was estimated at 1.6 to 3.5 and at 208 for H5N1 and H7N7 avian influenza strains respectively, and at 10.18 for an equine influenza strain [28,30-32]. In these studies *R *was reduced to 0 in chickens vaccinated with an inactivated H5N2 strain against an H5N1, and it was also 0 in chickens vaccinated with two inactivated H7N1 and H7N3 strains against an H7N7 avian flu virus. In contrast, a recent study reported that transmission of a H5N1 was not affected by vaccination of broiler chickens and suggested that this might have been due to the interference of maternal immunity [33]. In horses, *R *was reduced to 2.4 for a homologously vaccinated population and to 4.9 for a heterologously vaccinated population. To our knowledge this is the first study that has calculated *R*_0 _for influenza A virus in pigs and *R*_0 _is within the reported values for influenza in other species. In addition, results from our study also suggest that vaccination of pigs can be used to reduce their level of susceptibility, although a degree of virus spread can still take place in populations with immunity.

The reproduction ratio estimates obtained from the deterministic model provide useful threshold values and rates, while stochastic models allow one to investigate estimated outcomes and probabilities such as the number of new cases. Stochasticity is important to consider when dealing with small populations, such as the population reflected in this experimental study. Stochastic models can also be used to complement deterministic models and to assess whether major inferences are comparable across the two different approaches.

From the deterministic models, *R*_0 _estimates for the NV replicates ranged from 8.51 to 19.81. Therefore, following the introduction of one typical infected pig into a completely susceptible population, 9 to 20 secondary cases can be expected due to the typical infectious pig during its entire infectious period. The NV stochastic models reiterate this estimate by showing that major influenza virus outbreaks are likely to occur in small naïve populations following the introduction of one infected pig. The distribution of new cases was also similar across replicates for the NV group with either small or large outbreaks occurring. Once the initial infected pig transmitted the infection to a susceptible pig, the infection chain proceeded until all or nearly all animals were infected. These simulations likely describe high morbidity rates observed in field settings with susceptible animals. The NV stochastic models also clearly show that no new cases or minor outbreaks can also occur, albeit at low frequency, in a similar population even if *R*_0 _values are high.

The *R *estimates obtained from the deterministic models for the HE groups were lower than that of the NV groups and ranged from 0.32 to 1.8. The resultant stochastic models again reiterate the values obtained in the deterministic models. The HE replicates with lower *R *values (0.32 and 0.51) yielded a higher proportion of stochastic simulations with no new cases. This finding is expected as a lower *R *estimate equates to fewer secondary cases due to a typical infectious individual during its entire infectious period. In all HE replicates, the proportion of simulations with major outbreaks was low. On the contrary, no new cases were observed in at least 40% of the simulations regardless of the HE replicate. The proportion of simulations in which no new cases were observed was greatest for those replicates in which the *R *value was lower.

The impact of vaccination can be assessed in this small population from results of the stochastic models by comparing the NV and HE groups. In small, naïve populations, the introduction of an influenza virus infected pig resulted in a major outbreak and infected all susceptible animals in greater than 84% of simulations. Following vaccination with a HE vaccine in a small population, the proportion of simulations in which all susceptible animals become infected following the introduction of an infected pig was very small (< 7%). However, in the presence of HE immunity, transmission of influenza virus to susceptible animals did occur and it was not prevented in as high as 60% of the simulations as seen in HE replicate 2.

Antibodies against the hemagglutinin protein have been correlated with strong immune response to influenza and with a decrease of the likelihood of becoming infected. However, the levels of protective HI titers against virus replication are not easily established due to the continuous antigenic drift of the virus and due to that reciprocal HI titers alone may not guarantee immunity or predict susceptibility [13,52,53]. Kyriakis et al. [52], in an experimental study with pigs vaccinated with four different commercial vaccines and challenged with a heterologous H1N1 field isolate, found that pigs with reciprocal HI antibodies titers ≥ 20 against the field strain were virologicaly protected [52]. Van Reeth et al. determined that for complete virological protection against a heterologous strain, HI titers as high as 160 are required [13]. In those studies the viral protection was referred to individual virus titers from lung lobes at 3 to 4 days post experimental challenge and related to clinical signs, but not to transmission of the virus. In our study, two weeks after the second vaccine and before exposure to seeder pigs, pigs in the HO group had geometric mean HI titers of 295, while the geometric mean in the HE group was 14 against the challenge strain (maximum reciprocal titer was 80). The challenge virus demonstrated low serologic cross-reactivity with the antiserum induced by the heterologous vaccine and only nine of the forty pigs from the HE group had reciprocal HI titers of greater than 40 against the challenge strain before exposure to the seeder pigs. Of those, two pigs became infected and seven remained uninfected for the duration of the study. Therefore, HI titers against the challenge strain in HE pigs were not able to predict the infectious status of the pigs at the end of the study. The low immunogenicity against the challenge virus in the HE pigs was expected and is in agreement with other studies where pigs vaccinated with licensed inactivated vaccines became infected when challenged with heterologous influenza viruses [11,12,54,55]. At the end of our study not all pigs that had seroconverted to the challenge strain shed virus. Eighteen of the 40 pigs in the HE group had positive HI titers against the challenge strain at necropsy, and in 8 of the 18 pigs with HI titers ≥ 40 the virus could not be detected from nasal swabs samples. Lack of detection could be due to protection and lack of virus replication, or due to the amount of virus excretion being below detectable levels.

Another point of consideration was that our definition in regards to "homologous" and "heterologous" was related to the HA genetic and antigenic clustering. The challenge strain belonged to the beta influenza H1 cluster and strains in the commercial vaccine were from the gamma and delta clusters which shared 92.2% and 66.8% HA nucleotide similarity respectively with the challenge strain. Although it is known that cross-reactivity may exist between those clusters, this is considered limited [35] which is likely to influence the degree of infection. Therefore the outcome on transmission dynamics may also be influenced by the degree of cross-reactivity between clusters and further studies are needed to determine the extent of this.

Differences in transmission could also be due to differences in vaccine efficacy due to different antigen concentration, adjuvant used, or both factors [30,56]. A study with equine H3N8 strains documented the crucial role of vaccine dose and adjuvant in protection against heterologous challenge [57]. In our study, the amount of antigen was not standardized between the homologous and the heterologous vaccines because the HE group was vaccinated with a commercial licensed vaccine and information on the antigen content was not available. The improved protection provided by the autogenous vaccine was most likely due to its preparation with the virus strain homologous to the challenge virus. It is well documented that strain-specific antibody is more effective than cross reactive-antibody in conferring protection against flu infection [55]. In our study, pigs that received the homologous vaccine had no detectable virus in nasal secretions, had a robust antibody response to the challenge virus, and had significantly lower macroscopic lung lesion scores compared with the pigs that received the licensed heterologous vaccine. There were no differences between microscopic lesions between vaccinated groups, and histopathology in the NV and HE group was compatible with previous influenza infection. Histopathology for the HO group indicated that although lesions were absent in most of the homologous pigs, a few pigs had minimal bronchiolar lesions that were consistent with recovery from influenza infection. Despite the fact that all RT-PCR results in the HO group were negative, we cannot conclusively rule-out the possibility that infection took place in a few pigs in the HO group. If infection had taken place, infection appeared limited to the lower respiratory tract and virus could not be detected with the methods used in this study.

In addition, clinical signs were relatively mild in all the infected pigs, which is similar to what has been reported in previous studies using the same influenza virus [12,13,36,53]. In our study, both vaccines decreased the presence of disease and the impact of influenza infection. More importantly this study highlights the potential for influenza transmission despite the reduction of obvious clinical signs in partially immune populations and does not recommend the use of surveillance programs based on clinical signs in particular in partial immune populations. Moreover, as discussed previously, the virus can remain for extended periods of time in partially immune populations. Such a silent spread of influenza virus can lead to higher probabilities of transmission to other herds and people as also shown by Savill et al [58].

Overall, an important consideration in regards to inactivated vaccines is whether viruses can replicate and transmit in the presence of some degree of immunity. Under those conditions, virus escape variants can arise and can lead to antigenic drift, loss of vaccine efficacy, and emergence of new strains. Subclinical infections with virus shedding can occur in vaccinated animals, particularly when there is a mismatch between the vaccine and field virus strains [12,53]. In our study, virus replication and transmission was observed in the HE vaccinated group which suggest the potential role of vaccination in the establishment of enzootic infections and predisposition to virus change. However, whether virus changes happened as a result of vaccine immune pressure it is beyond the scope of this study and warrants future studies.

A limitation of our study is the inoculation dose of the seeder pigs that could lead to a higher shedding of influenza and subsequent overestimations of *R *compared to conditions that occur in the field. This study was also limited to one licensed vaccine and one autogenous vaccine and the results only reflect the transmission of a single H1N1 flu strain in a limited population. Therefore, transmission parameters in populations vaccinated with others vaccines and infected by different strains would need to be evaluated to further assess influenza transmission patterns in pigs. Additionally, the starting conditions of this study (S = 10, I = 1, r = 0) were designed to reflect a potential field scenario in which an infected pig was introduced into a pen of susceptible or vaccinated pigs. The parameters estimated in this study may not accurately reflect transmission parameters in differing population structures and settings.

In conclusion, transmission parameter estimates derived from this study can be used to further understand influenza transmission dynamics in naïve and immune swine populations. In our study, transmission to contact pigs was significantly reduced by vaccination but it could not be completely prevented when a heterologous vaccine was used. Virus transmission and replication was delayed and variable in the heterologous vaccinated group. On the other hand, transmission could not be detected in homologous vaccinated pigs. In the heterologous vaccinated group, active transmission took place even in the absence of clinical signs and presence of immunity, resulting in the silent spread of influenza virus which may contribute to the establishment of enzootic infected populations and increased probabilities of transmission to other herds. Such populations can in turn also be a risk for interspecies transmission and zoonotic infections. The results of the present study support field observations regarding the variability observed with influenza vaccines to affect transmission and spread of influenza virus.

## Competing interests

The authors declare that they have no competing interests.

## Authors' contributions

AR was a MS student and was involved in the animal experiment, statistical analyses, evaluation of results and preparation of the draft manuscript. MA was a PhD candidate and was involved in the animal experiment, statistical analyses, and preparation of the manuscript. MG was involved in experimental design, virus strain selection and diagnostic support. HSJ was involved in autogenous vaccine preparation and serologic data analyses. JD was involved in the study design, statistical analysis and interpretation of the results. SD was involved in preparation of IACUC protocols, histopathology evaluation and lesion scores. MT secured funding, was involved in study design, study execution, interpretation of results and had final responsibility for the study. All authors read and approved the manuscript.
